# The Evolving Burden of Stroke in China’s 832 Poverty-Alleviated Counties (2019-2024): Nationwide Spatiotemporal Analysis

**DOI:** 10.2196/91487

**Published:** 2026-06-03

**Authors:** Mengdi Chen, Dong Xia, Wenping Li, Jie Wang, Zhiyu Lv, Jiapeng Chen, Lulu Zhang

**Affiliations:** 1 Department of Health Services, Naval Medical University, 800 Xiangyin Rd, Shanghai, 200082, China, 86 81871421; 2 China Population and Development Research Center, Beijing, China

**Keywords:** stroke, poverty-alleviated counties, spatial analysis, incidence, prevalence, China

## Abstract

**Background:**

Stroke remains a leading cause of death and disability in China. Despite the nationwide poverty alleviation being achieved by 2020, residents of poverty-alleviated counties continue to experience poorer health outcomes. There is a critical knowledge gap regarding the evolving spatiotemporal patterns of stroke burden in this large postpoverty population.

**Objective:**

This study aimed to quantify the longitudinal trends in stroke prevalence and incidence across all 832 poverty-alleviated counties in China from 2019 to 2024, stratified by age and sex; to characterize the spatiotemporal evolution and geographic clustering of the stroke burden; and to compare this burden in poverty-alleviated counties with that of the general Chinese population during the key transitional period from 2019 to 2021.

**Methods:**

We conducted a nationwide retrospective analysis using data from China’s National Health Poverty Alleviation Dynamic Management System (NHPADMS), covering 56.08 million residents. Temporal trends were analyzed using Joinpoint regression to calculate the annual percentage change and average annual percentage change (AAPC). Spatial clustering was assessed using Global and Local Moran *I*. Comparative analysis used age-standardized prevalence rates, referenced against the Global Burden of Disease standard population.

**Results:**

Stroke prevalence surged markedly from 65.18 to 359.60 per 100,000 population (overall AAPC: +22.95%), with the steepest increase among adults aged 20 to 39 years (AAPC: +35.63%). Stroke incidence remained relatively stable from 2020 to 2023 but declined notably in 2024. While absolute prevalence remained lower, its growth rate in poverty-alleviated regions (+61.85%) far exceeded that in the nonpoverty-alleviated areas (+3.82%) during 2019 to 2021. Spatial analysis revealed persistent hyperendemic clusters in northern provinces (notably the Hebei-Heilongjiang-Jilin-Inner Mongolia corridor) and a significant north-south gradient.

**Conclusions:**

The stroke burden in China’s poverty-alleviated counties increased sharply in recorded prevalence, exhibited a worrying shift toward younger adults, and became geographically polarized. Economic advancement has not translated into proportionate health gains. These findings underscore the urgent need for precision public health interventions that are geographically targeted and demographically tailored to address this evolving epidemic.

## Introduction

Stroke ranks as the second leading cause of death globally, surpassed only by ischemic heart disease [1]. In China, it remains the leading cause of adult mortality and disability, exhibiting a persistently rising prevalence [2,3]. Notably, rural Chinese populations demonstrate significantly elevated stroke incidence and mortality rates among low-income groups compared to their higher-income counterparts [4].

Over the past decade, China has prioritized poverty eradication, achieving nationwide poverty alleviation across all 832 nationally designated impoverished counties by the end of 2020. Nevertheless, the health status of residents in these newly lifted-out-of-poverty counties continues to trail behind the national average [5]. Despite nationwide prevention initiatives and efforts to promote equitable health care access under the “Healthy China 2030” framework [6], effective burden reduction remains unachieved, particularly among vulnerable populations [7,8].

Prior epidemiological studies on stroke among China’s poverty-alleviated populations have significant limitations: they often lack adequate population bases or are constrained by narrow scopes, such as restricting age ranges (eg, 35‐64 y), exhibiting selection biases, relying on small and nonnationally representative samples, focusing solely on national or provincial scales, or being confined to periods during prealleviation poverty [9-13]. Critically, most foundational studies were conducted 10 to 30 years ago. Furthermore, China has undergone rapid health transitions and profound sociodemographic shifts during the poverty alleviation period [14]. Marked rises in the prevalence of hypertension, smoking, overweight or obesity, and diabetes represent contemporary shifts in key modifiable risk factors, potentially altering the stroke burden [15-17]. This creates a knowledge gap regarding how the burden of stroke has evolved among these populations following the elimination of absolute poverty, especially during the post-2020 transition studies [18,19].

To address this gap, our nationwide investigation conducts spatially explicit, age-stratified, and sex-stratified analyses of stroke incidence and prevalence trends across China’s 832 poverty-alleviated counties from 2019 to 2024. This granular methodological approach distinctly identifies microscale geographic disparities typically obscured in aggregated data, thereby establishing a robust epidemiological foundation for designing precision interventions targeting high-risk clusters. This study aims to answer 3 core research questions: (1) How have the prevalence and incidence rates of stroke evolved longitudinally in poverty-alleviated counties, and do these trends exhibit variations by age, sex, or region? (2) What characterizes the spatiotemporal evolution of the stroke burden during the study period, including spatial clustering and regional diffusion patterns? (3) How does the stroke burden in poverty-alleviated counties during the transition period (2019‐2021) compare with the concurrent burden across contemporary China?

## Methods

### Data Source

Incidence, prevalence, and population data (2019‐2024) for the poverty-alleviated population were systematically extracted from China’s National Health Poverty Alleviation Dynamic Management System (NHPADMS), maintained by the China Population and Development Research Center. The database encompasses 56.08 million officially registered poverty-alleviated residents across 832 county-level administrative divisions that completed national poverty eradication. Stratification followed NHPADMS age groups: 20 to 39, 40 to 64, and 65 years and older. The annual denominator population consists of these poverty-alleviated residents, which changes dynamically due to death, migration, or birth. The definition of “poverty-alleviated population” is provided by the National Administration for Rural Revitalization of China. The inclusion of “stroke” cases operates under the policy framework established by the National Health Commission of China, which lists stroke as a key disease for specialized treatment in poverty–alleviation health care initiatives. Consequently, all registered cases meet China’s current clinical diagnostic criteria for cerebrovascular diseases. Stroke was diagnosed using National Clinical V.2.0 of the International Classification of Diseases, Tenth Revision, disease codes. Case ascertainment relied on passive registry linkage. As health-focused poverty-alleviation efforts have progressed, primary health care facilities in poverty-alleviated counties have carried out systematic screening and registration for key diseases, including stroke, which may have led to a significant increase in the detection rate of cases.

The county-level administrative boundaries used in this study were sourced from the National Geomatics Center of China (NGCC) [20]. All spatial analyses and the resulting distribution maps were generated using the unmodified standard basemaps provided by the NGCC (Map Audit number GS[2024]0650).

Comparative prevalence data for the general population of mainland China (2019‐2021) were obtained from the Global Burden of Disease (GBD) 2021 study (Institute for Health Metrics and Evaluation) [3].

### Temporal Analysis

Temporal trends (2019‐2024) in stroke prevalence and incidence were quantified using the Joinpoint regression model (Joinpoint Regression Program v5.2.0, National Cancer Institute). The optimal number of joinpoints (turning points) was selected using the Weighted Bayesian Information Criterion (WBIC), which is the default model selection method in Joinpoint version 5.0 and higher. WBIC combines the classical Bayesian Information Criterion (BIC) and the BIC3 (a modified BIC with a harsher penalty) using a data-driven weight based on the partial *R*^2^, thereby adapting to the magnitude of slope changes in the data. This approach does not require manual specification of the maximum number of joinpoints. In Joinpoint regression, the maximum number of joinpoints is inherently constrained by the number of time points; for a 6-year series, the algorithm considers models with up to 3 joinpoints. The model identifies significant temporal turning points (joinpoints), representing years when the trend (eg, prevalence or incidence) slope undergoes a statistically significant change [21]. These turning points reflect shifts in disease dynamics, potentially linked to factors like public health measures or environmental exposures. The annual percentage change (APC) was calculated for each trend segment identified by the selected model, and the average annual percentage change (AAPC) was computed as a summary measure for the entire study period, with 95% CI estimated through the empirical quantile method.

The statistical analysis used the *χ*^2^ test to assess differences in rates (incidence or prevalence) across groups (age and sex strata), with pairwise comparisons adjusted using the Bonferroni correction (*α*=.05).

### Spatial Analysis

Global and local spatial autocorrelation analysis was performed at the county level to identify the geographic clustering of incidence using ArcGIS 10.8 [22]. A spatial weights matrix was constructed based on the Inverse_Distance conceptualization, which defines the spatial relationship between counties as a function of the inverse distance between their geographic centroids. To ensure the robustness of our findings, we conducted a sensitivity analysis by varying the distance threshold. Specifically, we compared results using 3 distance thresholds: (1) the default threshold automatically determined by ArcGIS, (2) a smaller threshold (50% of the default), and (3) a larger threshold (200% of the default). The statistical significance was determined using a permutation approach with 999 repetitions. For global analysis, we applied Global Moran *I* to assess the overall spatial pattern across the study area [23]. Values approximate −1 (dispersed) to 1 (clustered). A significantly positive Moran *I* (statistical significance was set at *P*<.05 for all analyses) indicates a clustered pattern where similar values are spatially aggregated, whereas a significantly negative value suggests a dispersed pattern. A value near zero implies a random spatial distribution. For local analysis, to pinpoint the specific locations and types of clusters, we conducted a Local Indicators of Spatial Association analysis using Anselin Local Moran *I* [24]. This method identifies statistically significant (statistical significance was set at *P*<.05 for all analyses) spatial units belonging to 4 categories: high-high clusters (a high-incidence county surrounded by other high-incidence counties, ie, a hotspot), low-low clusters (a coldspot), and spatial outliers (high-low or low-high units).

### Comparative Analysis With National Data

To control for the influence of differing population age structures on comparability, this study used the direct age standardization method to calculate the stroke prevalence rate. The GBD world population age standard was used as the reference population structure [25]. The key standardized metric, along with its definition and computational formula, is as follows:

Age-standardized prevalence rate (ASPR) represents the prevalence rate adjusted to a standard age distribution. It reflects the stroke prevalence that would be observed if the populations being compared shared an identical age structure, allowing for valid comparisons across groups with different age compositions.


(1) $$ASPR=\left(\frac{\sum Age-Stratum\;prevalent\;cases}{Total\;population}\right)\times \lambda_{\left[age\right]}\times 100,000$$

λ is the standardized proportion according to the GBD world population age standard.

The comparative analysis focused on the years 2019 to 2021 because this period captured the most dramatic initial surge in age-standardized stroke prevalence within the poverty-alleviated population, increasing from 65.18 to 172.79 per 100,000 population. This steep rise established a new baseline for subsequent trends. To determine whether this rapid increase reflected a phenomenon specific to this recently poverty-alleviated group or was part of a broader national pattern, we compared the magnitude and pace of prevalence change with that of the nonpoverty-alleviated regions (defined as the population of China excluding those residing in poverty-alleviated regions) during the same 3-year window. This period also coincided with the conclusive phase of China’s targeted poverty alleviation campaign (ending in 2020) and the onset of the pandemic, providing crucial context for interpreting shifts in health care engagement and data reporting.

### Ethical Considerations

This study involved a secondary analysis of deidentified national public health records. It was reviewed and confirmed to be exempt from full ethical review by the Naval Medical University Ethics Committee.

## Results

### Overall Trends in Prevalence and Incidence

From 2019 to 2024, the overall prevalence of stroke demonstrated a marked and sustained increase, rising from 65.18 to 359.60 per 100,000 population. The incidence rate remained relatively stable through 2023 before a notable decline in 2024. Joinpoint regression quantified a significant upward trend in prevalence for the overall population, with an AAPC of +22.95% (*P*=.006). This increasing trend was consistent across all subgroups but varied in magnitude: the most rapid rise was observed in the 20 to 39 years age group (AAPC=+35.63%, *P*<.001), followed by the 40 to 64 years (AAPC=+28.29%, *P*<.001), and the 65 years and older age groups (AAPC=+26.42%, *P*<.001). In the 20 to 39 years age group, prevalent stroke cases rose from 173 (1.07 per 100,000 population) in 2019 to 1728 (10.65 per 100,000 population) in 2024, with the absolute increase accounting for 0.90% of all new cases during the same period. Although the AAPC was highest in this group, the absolute burden remained modest. When stratified by sex, the AAPC was slightly higher for females (+24.31%, *P*<.001) than for males (+22.16%, *P*<.001; Table 1).

**Figure 1.**
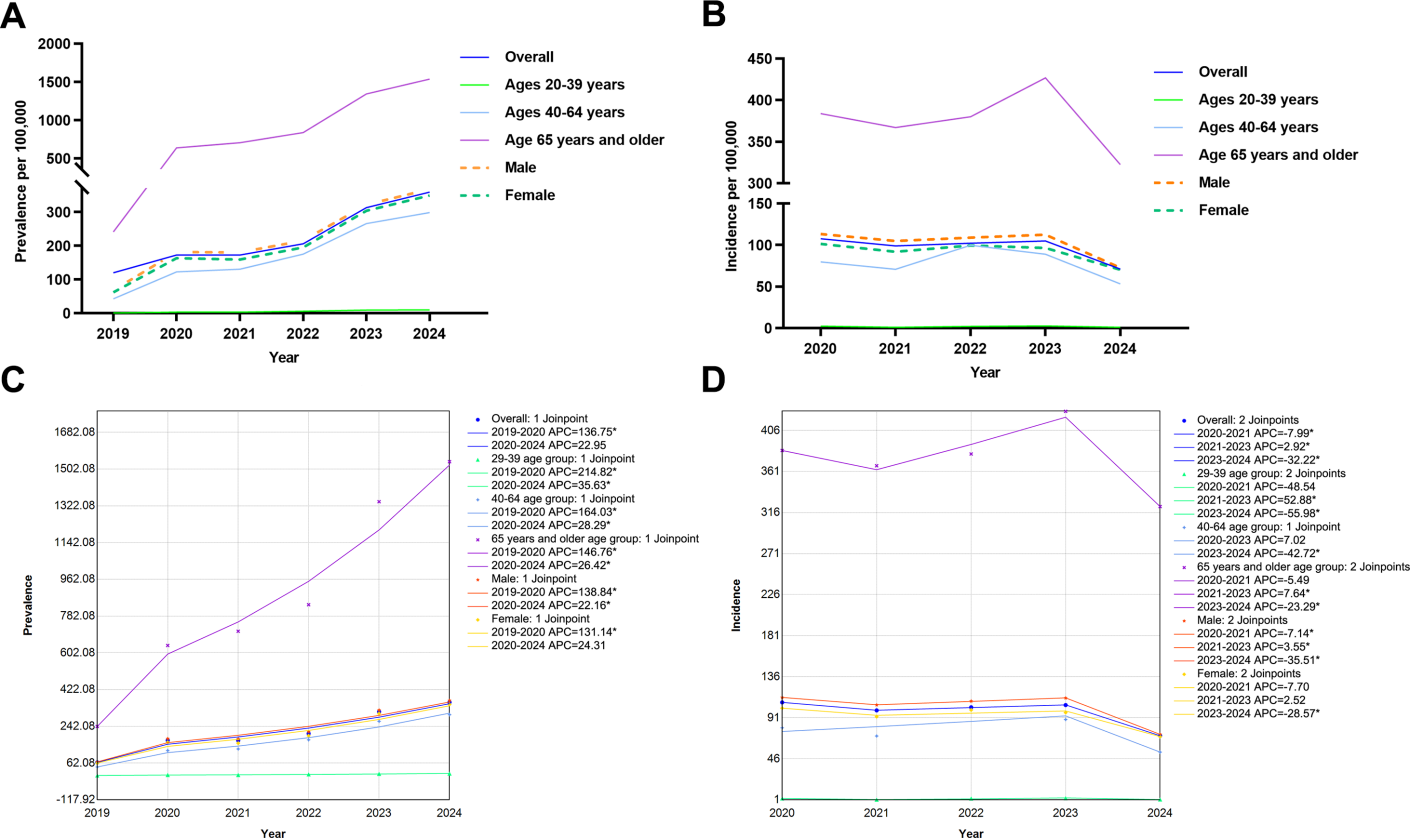
Trends in prevalence and incidence rates by age and sex groups among poverty-alleviated populations. (A) Trends in prevalence rates, 2019‐2024. (B) Trends in incidence rates, 2020‐2024. (C and D) Joinpoint regression analysis of stroke prevalence and incidence rates by sex and age groups among poverty-alleviated populations. Annual percentage change (APC) in prevalence rates (C) and incidence rates (D). In panels A and B, solid lines represent age groups (ages 20‐39, 40‐64, and 65 years and older) and dashed lines represent sex groups (male, female). Data are presented as rates per 100,000 population based on the registered poverty-alleviated population (n=56.08 million) across 832 counties. In panels C and D, * indicates that the APC value is statistically significant (*P*<.05).

For overall stroke incidence, the WBIC selected a model with 2 joinpoints (2021 and 2023). Consequently, 3 trend segments were identified: 2020 to 2021 (APC=−7.99%, *P*<.001), 2021 to 2023 (APC=+2.92%, *P*<.001), and 2023 to 2024 (APC=−32.22%, *P*<.001). The steepest decrease occurred in the last year of the observation period. Cross-sectional analyses revealed consistent patterns within each individual year from 2019 to 2024. Specifically, in every year observed, both stroke prevalence and incidence increased with advancing age, from the 20 to 39 years to the 65 years and older age groups. Additionally, the rates for both metrics were consistently higher in males than in females within each annual cross-section. The most pronounced year-on-year increase in prevalence occurred between 2019 and 2020, whereas the largest annual decrease in incidence was observed between 2023 and 2024 (Figure 1).

To assess whether the observed 2024 incidence decline disproportionately influenced overall trend estimates, we repeated the Joinpoint regression analysis after excluding 2024 data (ie, using only 2020‐2023 data). The overall incidence trend without 2024 remained stable and nonsignificant, confirming that the sharp decline in 2024 was not a statistical artifact that altered the long-term trend interpretation. In contrast, the prevalence trend without 2024 remained strongly positive (AAPC=+39.34%, 95% CI 16.47%‐65.40%, *P*<.001), consistent with the primary analysis. These results are presented in Multimedia Appendix 1.

**Table 1.** Stroke incidence and prevalence per 100,000 individuals (2020‐2024).^a^

Stroke incidence and prevalence	Year						AC^b^	AAPC^c^, % (95% CI)
	2019	2020	2021	2022	2023	2024		
Incidence	N/A^d^	107.80	99.02	102.43	104.87	71.21	−36.59	N/A
Incidence by sex								
Male	N/A	113.25	105.04	109.18	112.61	72.72	−40.53	N/A
Female	N/A	101.45	92.09	99.47	96.66	70.30	−31.15	N/A
*χ*^2^ (df)	—^e^	173^f^ (1)	228^f^ (1)	125^f^ (1)	327^f^ (1)	11^f^ (1)	—	—
Incidence by age								
20‐39 y	N/A	2.55	1.20	2.43	2.78	1.35	−1.20	N/A
40‐64 y	N/A	79.93	91	99.75	88.99	53.28	−26.65	N/A
≥65 y	N/A	384.01	367.29	380.17	426.96	322.54	−61.4	N/A
*χ*^2^ (df)	—	33,100^f^ (2)	79,573^f^ (2)	73,819^f^ (2)	66,903^f^ (2)	70,905^f^ (2)	—	—
Prevalence	65.18	173.11	172.79	206.63	313.74	359.60	+294.42	22.95^g^ (4.72‐43.73)
Prevalence by sex								
Male	67.86	181.43	180.19	215.54	321.37	368.52	+300.66	22.16^g^ (4.65‐42.05)
Female	62.08	163.44	159.57	195.74	304.24	349.36	+287.28	24.31^g^ (3.01‐49.41)
*χ*^2^ (df)	68^f^ (1)	250^f^ (1)	348^f^ (1)	258^f^ (1)	127^f^ (1)	139^f^ (1)	—	—
Prevalence by age								
20‐39 y	1.07	3.64	3.89	6.54	9.43	10.65	+9.58	35.63^g^ (24.28‐47.51)
40‐64 y	42.85	123.33	131.01	175.90	266.41	299.01	+256.16	28.29^g^ (10.41‐48.71)
≥65 y	241.48	637.86	707.68	837.58	1342.21	1538.02	+1296.54	26.42^g^ (10.91‐41.60)
*χ*^2^ (df)	54,201^f^ (2)	137,355^f^ (2)	156,901^f^ (2)	170,572^f^ (2)	274,136^f^ (2)	131,100^f^ (2)	—	—

a Stroke incidence and prevalence are based on the number of incident cases and the overall poverty-alleviated population.

b AC: absolute change.

c AAPC: average annual percentage change. Time periods for the AAPC analysis: prevalence (2020-2024).

d N/A: not applicable.

e Not applicable (eg, *χ*² values for AC and AAPC columns) or data not available (eg, incidence for 2019).

f The *χ*2 test result for between-group (age and sex) comparison of rates is statistically significant at *P*<.001.

g The AAPC is significantly different from zero at the *α*=.05 level. The AAPC in prevalence rates (2020-2024) was statistically significant across all subgroups, although the AAPC for incidence rates (2020-2024) was not.

### Temporal Distribution by County

**Figure 2.**
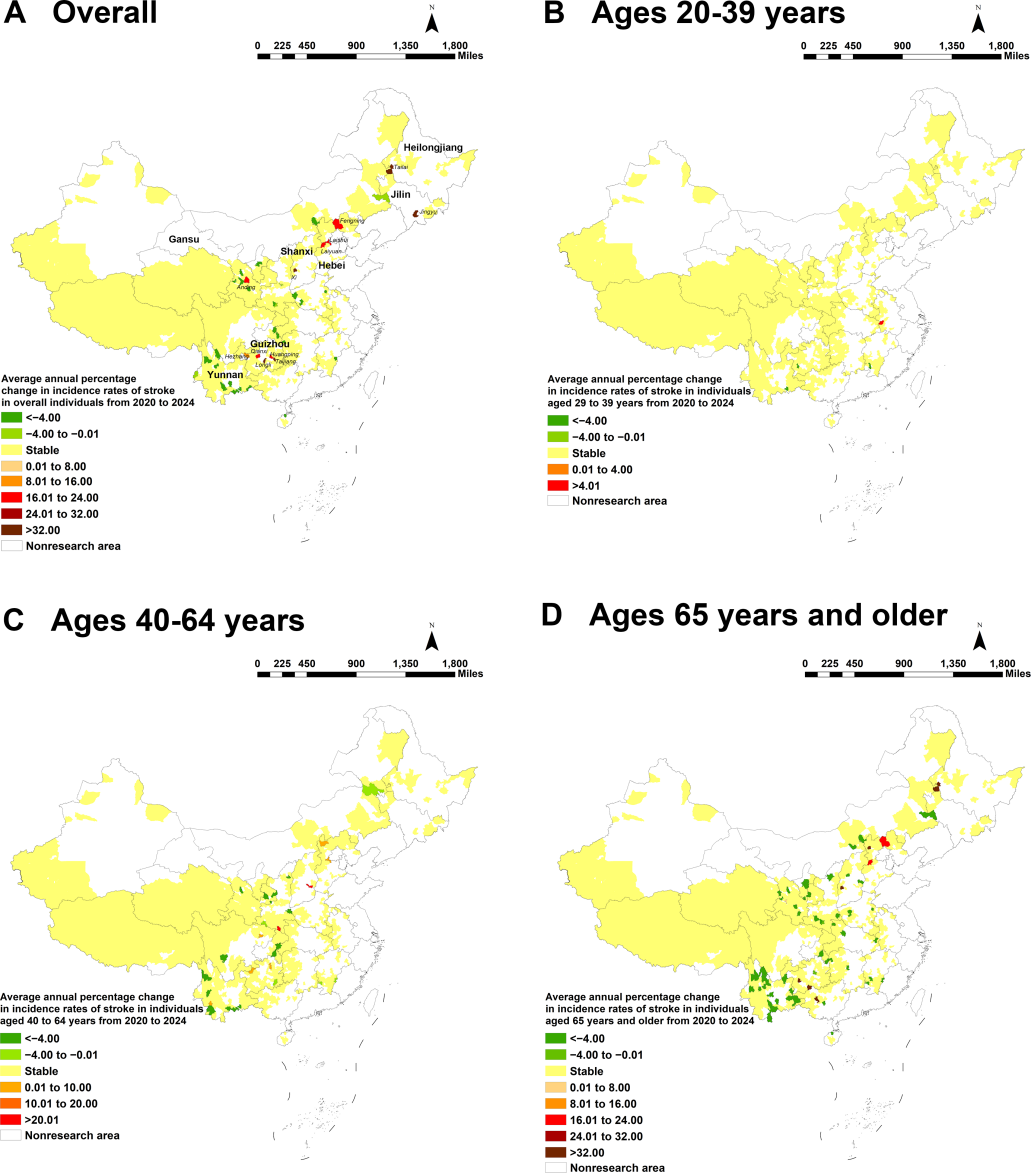
The average annual percentage change (AAPC) in stroke incidence among poverty-alleviated counties in China, 2020‐2024. (A) AAPC of stroke incidence in the overall population at the county level. The 12 counties highlighted in red exhibited a statistically significant upward trend (*P*<.05) and are annotated with their respective county and province names. (B-D) AAPC of stroke incidence stratified by age group: 20‐39 years (B), 40‐64 years (C), and 65 years and older (D). White areas represent counties that were never designated as impoverished and thus are not poverty-alleviated counties; these counties are excluded from the analysis of this study.

Temporal trends in stroke incidence were analyzed in 832 poverty-alleviated counties between 2020 and 2024. Figure 2 presents the AAPC in incidence rates by county. Among these counties, 1.4% (n=12) showed a rising trend in stroke incidence, while 3.4% (n=28) exhibited a declining trend. No statistically significant upward or downward trends in incidence rates were observed in the remaining counties (AAPC=0, *P*>.05). The 12 counties demonstrating upward trends were distributed across 6 provinces: 5 in Guizhou (Qianxi, Hezhang, Huangping, Taijiang, and Longli), 3 in Hebei (Laishui, Laiyuan, and Fengning), and 1 each in Shanxi (Xi), Jilin (Jingyu), Heilongjiang (Tailai), and Gansu (Anding). In contrast, counties with declining trends showed a pronounced concentration in Yunnan Province in southern China, which accounted for 10 of the 28 counties.

Age-stratified analysis indicated that increasing trends were primarily observed in older age groups, with 8 and 9 counties showing rises in the 40 to 64 and 65 years and older age groups, respectively, compared to only one county in the 20 to 39 years age group. Notably, within the 65 years and older age group, Yunnan Province also had the highest number of counties (n=20), exhibiting a declining incidence trend (Figure 2).

**Figure 3.**
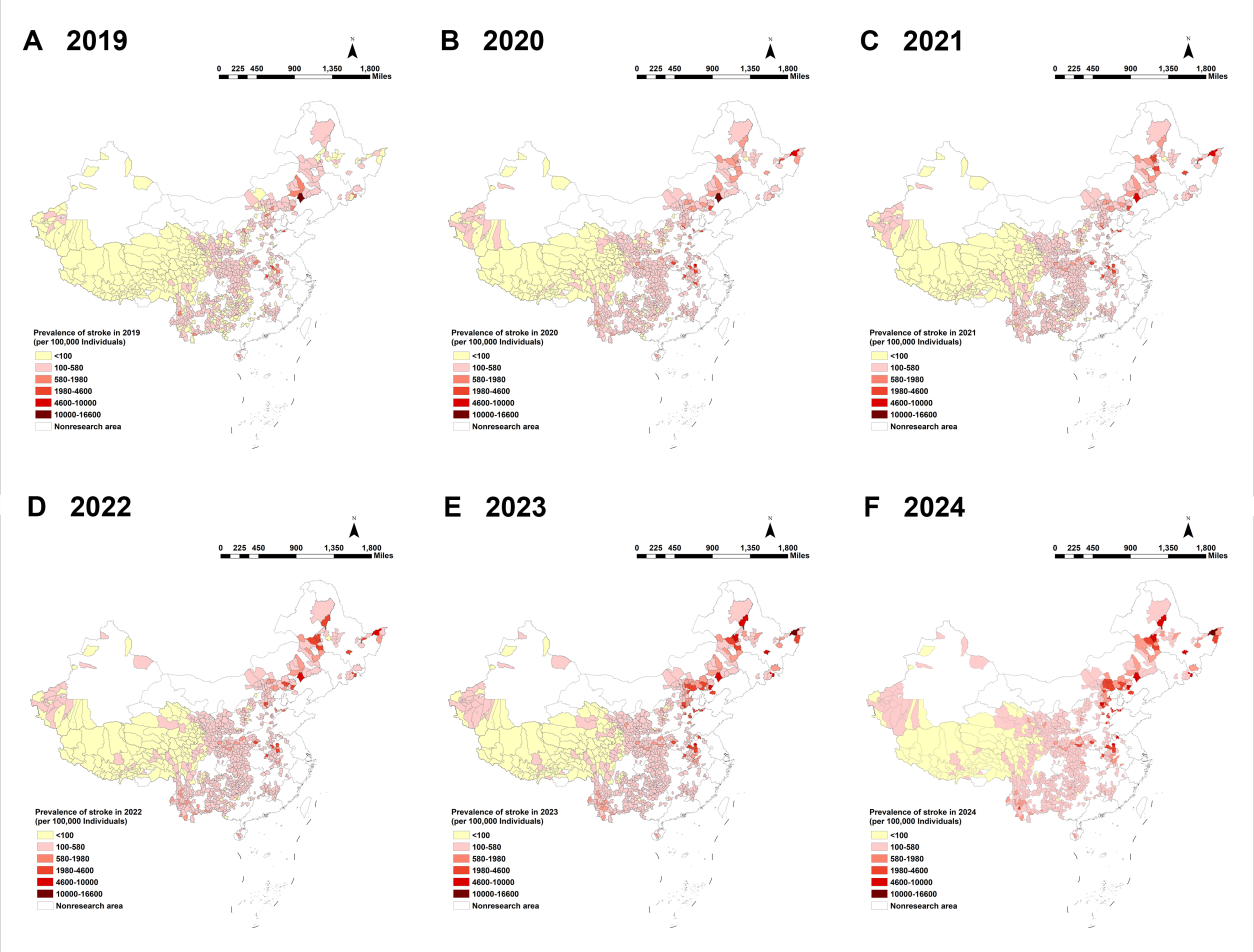
Spatiotemporal distribution of stroke prevalence among 832 poverty-alleviated counties in China, 2019‐2024. (A-F) Spatial distribution of age-standardized stroke prevalence (per 100,000 population) across 832 counties for each year from 2019 (A) to 2024 (F). White areas represent counties that were never designated as impoverished and thus are not poverty-alleviated counties; these counties are excluded from the analysis of this study.

**Figure 4.**
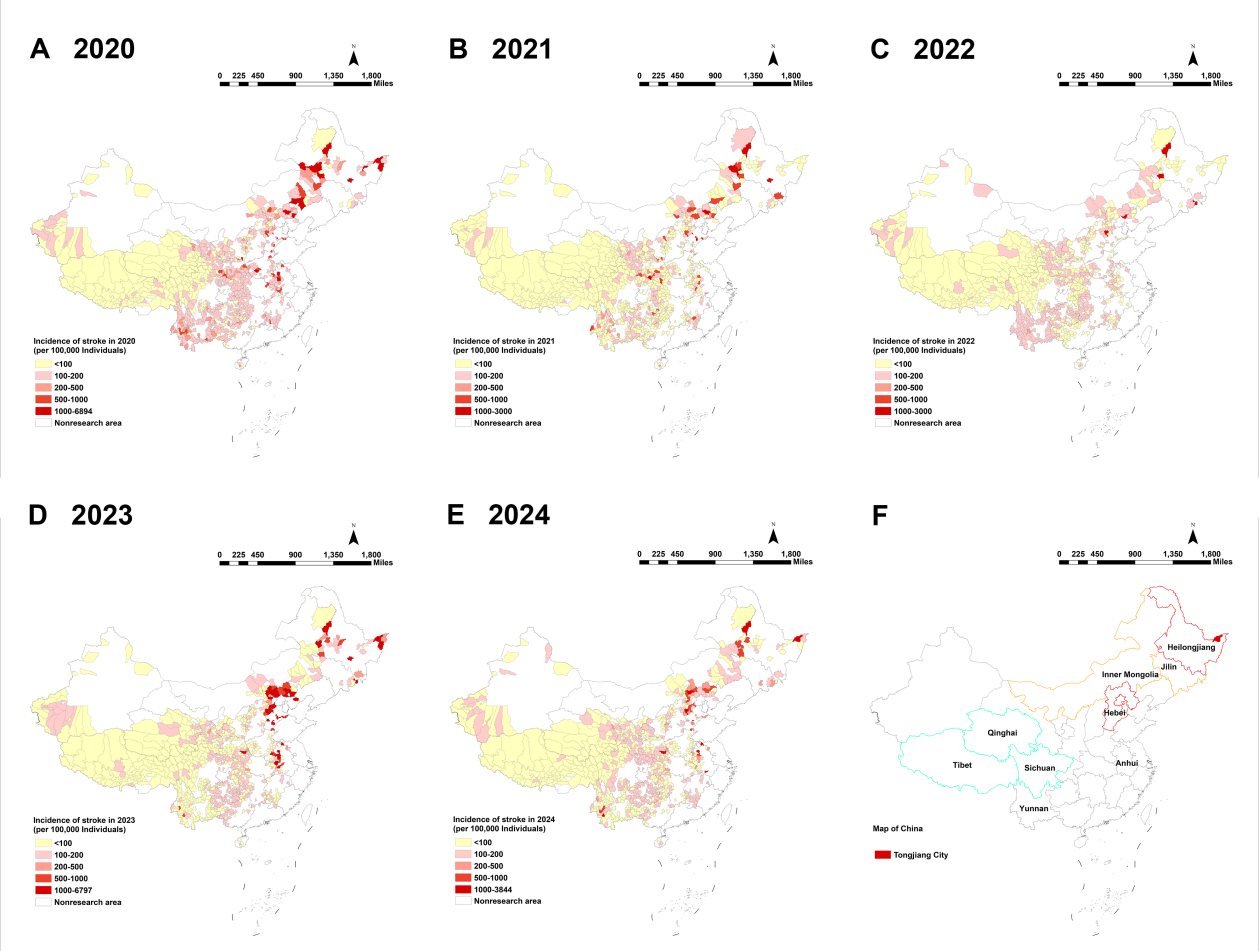
Spatiotemporal distribution of stroke incidence among 832 poverty-alleviated counties in China, 2020‐2024. (A-E) County-level stroke incidence rates (per 100,000 individuals) for each year from 2020 (A) to 2024 (E). (F) A summary map synthesizing spatial patterns across the study period. Provinces are categorized based on the persistent clustering of incidence: high-incidence provinces (eg, Hebei, Heilongjiang, Jilin, Inner Mongolia, and Anhui) and low-incidence provinces (eg, Qinghai, Sichuan, Tibet, and Yunnan). Tongjiang City (highlighted in red), Heilongjiang Province, is highlighted for recording both the highest incidence and prevalence rates in 2024. A pronounced north-to-south decreasing gradient is evident, with hyperendemic clusters concentrated in the northern Hebei-Heilongjiang-Jilin-Inner Mongolia corridor. White areas represent counties that were never designated as impoverished and thus are not poverty-alleviated counties; these counties are excluded from the analysis of this study.

The spatiotemporal analysis from 2019 to 2024 revealed a clear and persistent geographical hierarchy in stroke burden across China’s poverty-alleviated counties: high in the north, low in the south, with the most severe concentrations localized in the northeast and north China regions. Counties with the highest prevalence clustered consistently within a northern corridor encompassing Hebei, Heilongjiang, Jilin, and Inner Mongolia, followed by Anhui Province in eastern China. This spatial pattern remained stable throughout the 6-year observation period, during which stroke prevalence rates showed a marked overall increase (Figure 3). The distribution of incidence from 2020 to 2024 mirrored this pattern, with high-incidence counties concentrated in the north (eg, Hebei, Heilongjiang, Inner Mongolia, and Jilin) and low-incidence clusters in the southwest (eg, Sichuan and Qinghai). In 2024, among approximately 54 million residents of poverty-alleviated counties, 25,458 incident stroke cases and 194,911 prevalent cases were recorded. The highest incidence and prevalence rates nationwide were observed in Tongjiang City, Heilongjiang Province, underscoring the heightened burden in these high-burden northern regions (Figure 4).

### Spatial Distribution by County

The results of the global spatial autocorrelation analysis are presented in Table 2. All annual Global Moran *I* indices demonstrated positive values (range: 0.09‐0.30) with statistical significance (*P*<.001), indicating significant spatial clustering of stroke incidence in the general population. The Moran *I* exhibited progressive elevation from 2020 to 2023 (0.09→0.30), followed by a decline to 0.12 in 2024. Compared to the 20 to 39 years age group, both the 40 to 64 years and 65 years and older age cohorts exhibited higher and statistically significant Moran *I* values, revealing stronger spatial clustering of stroke incidence. An exceptional pattern emerged in the 2021 young-middle-aged cohort (20‐39 y), demonstrating nonsignificant spatial autocorrelation (Moran *I*=0.003, *z*=0.64, *P*>.05). Both sex groups exhibited significant spatial clustering across all years (*P*<.001), though males displayed marginally higher Moran *I* values than females (eg, 2023: 0.32 vs 0.26). The sensitivity analysis using alternative distance thresholds (50% and 200% of the default) yielded Global Moran *I* values that were highly consistent with those obtained using the default threshold (Multimedia Appendix 2), confirming the robustness of our spatial clustering findings.

Statistical significance was achieved for all study years (*P*<.001) except in the 20 to 39 years subgroup. Spatial clustering intensity increased from 2020 to 2023 but declined in 2024. Positive Moran *I* values (*I*>0) indicate spatial clustering where high-incidence counties cluster with adjacent high-incidence counties and low-incidence counties cluster with adjacent low-incidence counties. Values represent Moran *I* with corresponding 95% CI (lower-upper). *P* values were computed using Monte Carlo permutation tests (999 repetitions; significance threshold *α*=.01). For details on spatial weight matrix construction and sensitivity analysis, see *Methods* (*Spatial Analysis* section).

**Figure 5.**
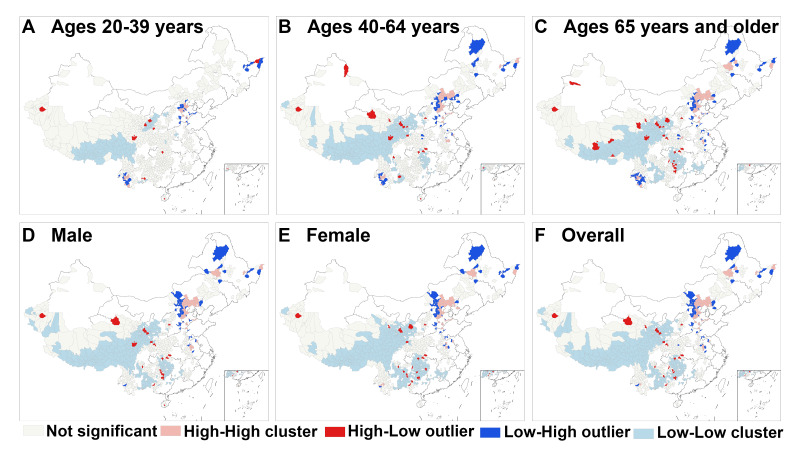
Local spatial clustering of stroke incidence among 832 poverty-alleviated counties in China, 2024. Local spatial clustering of stroke incidence in individuals aged 20‐39 years (A), individuals aged 40‐64 years (B), individuals aged 65 years and older (C), male (D), female (E), and overall population (F). Cluster types were classified using local Moran *I*: high-high: high-prevalence county surrounded by high-prevalence neighbors (hotspot area); low-low: low-prevalence county surrounded by low-prevalence neighbors (coldspot area); high-low: high-prevalence county surrounded by low-prevalence neighbors (spatial outlier); low-high: low-prevalence county surrounded by high-prevalence neighbors (spatial outlier). White areas represent counties that were never designated as impoverished and thus are not poverty-alleviated counties; these counties are excluded from the analysis of this study.

We conducted a clustering and outlier analysis of stroke incidence rates across population subgroups in 832 counties that achieved poverty alleviation by 2024. The results revealed 4 distinct spatial clustering patterns in overall stroke incidence rates within these counties. High-high clusters predominantly emerged in northern Chinese cities, including Hebei, Inner Mongolia, and Heilongjiang, where stroke incidence significantly exceeded that of neighboring areas and was consistent across all age-sex groups, except for those aged 20 to 39 years. This pattern may indicate high-risk disease zones. Identifying these high-cluster zones is essential for developing targeted disease prevention and control strategies; therefore, we compiled a list of county-level high-cluster zones with elevated stroke incidence across all subgroups for 2024 (see Multimedia Appendix 3 for details). Low-low clusters were concentrated in extensive contiguous areas of central and western China, such as Sichuan, Qinghai, Yunnan, and Guizhou, where stroke incidence was markedly lower than in neighboring regions. High-low clusters were scattered throughout central and western China, where counties exhibited higher stroke incidence while neighboring counties reported lower rates, potentially reflecting localized disease hotspots. Low-high clusters were sporadically distributed in northern China, where these areas demonstrated lower stroke incidence compared to neighboring regions with higher rates, possibly representing transitional zones for disease transmission (Figure 5).

**Table 2.** Global (Moran *I*) index for spatial association of stroke incidence (2020‐2024).

Year	Overall			Ages 20‐39 y			Ages 40‐64 y			Ages ≥65 y			Male			Female		
	Moran *I*	*z* scores	Pattern	Moran *I*	*z* scores	Pattern	Moran *I*	*z* scores	Pattern	Moran *I*	*z* scores	Pattern	Moran *I*	*z* scores	Pattern	Moran *I*	*z* scores	Pattern
2020	0.09^a^	11.27	Clustered	0.06^a^	7.64	Clustered	0.08^a^	10.45	Clustered	0.09^a^	11.15	Clustered	0.09^a^	10.98	Clustered	0.09^a^	11.56	Clustered
2021	0.09^a^	11.31	Clustered	0.00	0.64	Random	0.08^a^	10.34	Clustered	0.07^a^	8.48	Clustered	0.10^a^	11.73	Clustered	0.08^a^	9.99	Clustered
2022	0.19^a^	24.31	Clustered	0.03^a^	6.65	Clustered	0.29^a^	34.88	Clustered	0.37^a^	43.64	Clustered	0.20^a^	24.78	Clustered	0.15^a^	19.16	Clustered
2023	0.30^a^	35.76	Clustered	0.43^a^	53.18	Clustered	0.34^a^	40.41	Clustered	0.33^a^	39.77	Clustered	0.32^a^	38.19	Clustered	0.26^a^	31.87	Clustered
2024	0.12^a^	15.16	Clustered	0.05^a^	7.93	Clustered	0.14^a^	17.57	Clustered	0.10^a^	12.06	Clustered	0.12^a^	14.70	Clustered	0.12^a^	15.46	Clustered

a The corresponding significance level is *P*<.001.

### Comparative Analysis

A comparative analysis between poverty-alleviated regions and nonpoverty-alleviated regions of China (2019‐2021) revealed stark differences in the temporal trend of stroke prevalence. From 2019 to 2021, the age-standardized prevalence of stroke increased markedly in poverty-alleviated regions, with an APC of +61.85% (95% CI 14.99%‐129.60%, *P*<.001). This rate of increase was considerably steeper than the concurrent trend observed in nonpoverty-alleviated regions (APC=+3.82%, 95% CI 2.31%‐5.40%, *P*<.001). This accelerating trend was most pronounced in the 20 to 39 years age group, where the APC reached +88.96% (*P*<.001) in poverty-alleviated regions, compared to a much more modest increase of +1.94% (*P*<.001) in the same age group in nonpoverty-alleviated regions (Table 3).

It is important to note that the absolute prevalence estimates between the 2 datasets are not directly comparable due to fundamental differences in data sources and population coverage (see *Limitations* section). Therefore, only the direction and magnitude of temporal change (APC) are presented for comparative purposes. The underlying absolute values are provided in Multimedia Appendix 4 for transparency.

**Table 3.** Annual percentage change (APC, 95% CI) of stroke prevalence in poverty-alleviated regions and China (2019‐2021).^a^

Region	Overall, % (95% CI)	Ages 20‐39 y, % (95% CI)	Ages 40‐64 y,% (95% CI)	Ages ≥65 y, % (95% CI)
China (overall)	1.64^b^ (0.32 to 3.03)	1.78^b^ (1.09 to 2.47)	1.57^b^ (0.67 to 2.50)	1.73^b^ (0.04 to 3.62)
Poverty-alleviated regions	61.85^b^ (14.99 to 129.60)	88.96^b^ (26.77 to 184.81)	73.62^b^ (22.90 to 147.48)	74.10^b^ (26.97 to 136.55)
Nonpoverty-alleviated regions	3.82^b^ (2.31 to 5.40)	1.94^b^ (1.11 to 2.77)	1.57^b^ (0.52 to 2.65)	0.96 (−0.72 to 2.83)

a The group labeled “Nonpoverty-alleviated regions” refers to data for China excluding 832 designated poverty-alleviated regions. Prevalence rates were age-standardized using the Global Burden of Disease 2021 reference population structure (Institute for Health Metrics and Evaluation) to ensure comparability.

b Statistically significant annual trend, with a *P*<.001 for the APC. The APC and its 95% CI were calculated using Joinpoint regression (National Cancer Institute, V.5.2.0). *P*<.001 indicates a statistically significant annual trend in prevalence. As the model failed to detect the inflection point, the average annual percentage change and APC values are identical.

## Discussion

### Principal Results

This study presents the first retrospective analysis of data from all 832 poverty-alleviated counties in China, identifying the spatiotemporal trends of stroke epidemiology from 2019 to 2024 and revealing significant disparities in disease burden across different age groups, sexes, and geographical regions. This represents the largest spatiotemporal analysis of postpoverty transition epidemiology, overcoming prior fragmentation.

The increased stroke burden in China’s poverty-alleviated population, with the heaviest burden observed in individuals aged 65 years and older, aligns with previously documented trends in rural areas [7,26]. Stroke prevalence surged significantly in poverty-alleviated counties, with the 20 to 39 years cohort exhibiting the steepest acceleration (AAPC=+35.63%)—a trend disproportionately exceeding concurrent national growth. The rising prevalence of stroke among young adults, particularly in the age group of 20 to 39 years, has become a notable concern, especially linked to rapid urbanization in low-income regions. Two interlinked mechanisms may explain this trend: (1) behavioral risks: these regions have been experiencing a surge in traditional vascular risk factors, including hypertension, diabetes, and dyslipidemia, which are commonly associated with urbanization, such as sedentary lifestyles [27] and changes in dietary habits due to increased access to processed foods and reduced physical activity levels. For instance, one study reported an alarming rise in traditional risk factors among young adults, emphasizing how lifestyle changes linked to urbanization contribute to this trend in stroke incidence [28-30]. (2) Health care access gaps. Socioeconomic factors, including decreased access to health care and health education, leave young adults vulnerable to undetected vascular risks. It has been observed that in low-income communities, awareness and response to stroke symptoms are often inadequate, which can lead to delayed treatment and worse outcomes [31,32]. Importantly, the outmigration of working-age populations may concentrate stroke-prone individuals (eg, those with disabilities or chronic illnesses) in impoverished counties [33,34]. These factors collectively signal a failure of current preventive strategies to adapt to postpoverty health challenges.

The decline in incidence in 2024 should be interpreted cautiously. This decline may reflect a combination of factors, including registration delays (cases from late 2024 not yet entered into the system), residual postpandemic changes in health care utilization, and possibly random variation. A sensitivity analysis excluding 2024 data (Table S4 in Multimedia Appendix 4) showed that the incidence trend from 2020 to 2023 remained nonsignificant, indicating that the sharp decline in 2024 did not fundamentally alter the overall conclusion of no sustained increase in incidence. Longer-term data are required to determine whether this decline represents a genuine epidemiological shift.

Though males retained higher absolute prevalence, females experienced faster escalation (+24.31% vs +22.16%), uncovering neglected vulnerabilities. Hypertension in pregnancy, postpartum hemorrhage, and changes in vascular biomarkers [35-37] in women during middle age are women-specific risk factors that increase the risk of stroke [38]. Women in rural areas and low-income families are more susceptible to chronic diseases such as hypertension and diabetes, which further increase the risk of stroke [39]. Women are more susceptible to stress, depression, and psychological factors that increase the risk of stroke [35,40]. Sex differences in health literacy also affect stroke prevention and management. For example, 32% of rural women recognize the risk of high blood pressure, while 48% of men do [41]. These findings demand sex-specific prevention frameworks [35].

The analysis reveals a distinct and persistent north-south divergence in stroke burden across poverty-alleviated counties. Stroke prevalence demonstrates hyperendemic clusters in northern regions (notably the Hebei-Heilongjiang-Jilin-Inner Mongolia corridor), with a clear north-to-south decreasing gradient—exemplified by Tongjiang City in Heilongjiang recording the peak prevalence intensity in 2024. Similarly, incidence forms geographically anchored high-high clusters across these northern provinces, contrasting sharply with expansive low-low belts spanning southwestern regions such as Sichuan, Yunnan, and Qinghai. Three regional drivers likely contribute to this pattern of elevated stroke burden and high incidence in northern China’s poverty-alleviated counties: (1) cold climate effects: prolonged indoor confinement during the harsh northern winters disproportionately affects residents in poverty-alleviated counties. This behavior correlates with elevated blood pressure [42] and increased exposure to particulate matter (PM2.5) from biomass heating [43], a common practice in these areas. (2) Dietary sodium overload: Northeastern diets average 11.2 g of salt per day [44], far surpassing the World Health Organization recommendation of 5 g—a key modifiable risk factor. This high sodium intake is a key modifiable risk factor that directly elevates blood pressure [2,45], creating a persistent and widespread driver of stroke risk within the local populace. (3) Health care delivery bottlenecks: Northeastern regions, such as Heilongjiang Province, have a relative lack of medical resources, especially in rural and remote areas, where medical conditions are poor and the poverty-alleviated population has insufficient awareness of stroke [46], leading to inadequate preventive and treatment measures. Despite the marked rise in prevalence, an important nuance emerges when examining incidence. Overall stroke incidence across the 832 counties remained largely flat throughout the study period until 2024. However, this aggregate stability masks localized intensification: significant rises were concentrated in only 12 counties, predominantly driven by populations aged 40 years and older. This pattern suggests that while the overall entry rate of new stroke cases was stable at the macro level, specific geographic clusters—particularly those with aging demographics—are experiencing a tightening grip of the epidemic. We hypothesize that the decline in Moran *I* in 2024 may reflect temporal variation in data reporting completeness or random fluctuation, but further observation is needed. This finding shifts the focus from a universal incidence surge to the identification of high-risk geographic and demographic pockets where primary prevention efforts may be failing or where demographic aging is exerting its strongest effect.

During the pivotal postalleviation transition (2019‐2021), a striking disparity in the temporal dynamics of stroke prevalence emerged between poverty-alleviated and nonpoverty-alleviated regions. While the recorded age-standardized stroke prevalence in poverty-alleviated regions increased at an annual rate of 61.85% over 2 years, the concurrent increase in nonpoverty-alleviated regions was only 3.82%. We caution that the absolute prevalence estimates from the 2 data sources are not directly comparable due to fundamental methodological differences (see Limitations section). Therefore, only the direction and magnitude of temporal change (APC) are comparable between the 2 populations; any comparison of absolute rates or inference about confounding factors is not valid. Nevertheless, the markedly faster pace of increase in poverty-alleviated regions suggests a distinct epidemiological trajectory during the postalleviation transition period. This disparity can be understood as a “catch-up” phenomenon driven by 2 concurrent forces. First, the conclusive phase of the national poverty alleviation campaign (ending in 2020) significantly enhanced primary health care access and systematic screening in these regions, leading to improved detection and reporting of preexisting, previously undiagnosed cases [47]. Second, despite improved access, a relative lag in population-level stroke awareness and health literacy persists [48]. This lag likely results in delayed health care–seeking for transient ischemic attacks or minor strokes, allowing many cases to progress to a more severe or recordable stage before diagnosis, thereby contributing to the rising incidence of recorded prevalent cases. Thus, the steep upward trend does not merely represent a convergence toward the level observed in nonpoverty-alleviated regions but reveals a critical phase during which previously unmet health care needs became visible. Importantly, this surge should be interpreted primarily as a “detection catch-up” of previously unregistered cases, rather than as a mere epidemiological explosion.

In conclusion, this nationwide spatiotemporally explicit analysis reveals that the stroke burden in China’s poverty-alleviated counties is not merely a residual challenge of the past but a dynamically evolving epidemic characterized by a sharp increase in recorded prevalence, alarming shifts toward younger adults, and stark geographical polarization. Our findings fundamentally recalibrate the postpoverty health narrative: economic advancement has not automatically translated into commensurate health gains. Instead, it may have unmasked or even accelerated latent epidemiological risks. The concentrated burden in northern “hotspots” and among the young working-age population signals specific failures in current primary prevention strategies. Therefore, moving beyond uniform, poverty-alleviation-era health policies is imperative. Future interventions must be geographically precision-targeted to the identified high-risk clusters—hotspot identification enables precision resource allocation under China’s Rural Revitalization Health Initiatives—and demographically tailored to address the unique vulnerabilities of young adults and women. This shift is crucial for mitigating the impending wave of noncommunicable diseases and achieving equitable health outcomes in the post-poverty alleviation era.

### Limitations

Despite its comprehensive scope, this study has several limitations that should be considered when interpreting the findings. First, a fundamental limitation is the inability to distinguish the extent to which the observed surge in stroke prevalence reflects a true increase in disease burden versus improved case ascertainment driven by poverty-alleviation policies. The national poverty-alleviation campaign (ending in 2020) substantially enhanced primary health care access and systematic screening in poverty-alleviated counties, likely leading to the detection and registration of previously undiagnosed prevalent cases. Consequently, the reported trends should be interpreted as a combination of genuine epidemiological change and policy-induced detection improvement. Second, reliance on retrospective surveillance data may introduce variability in diagnostic accuracy and case ascertainment across different counties and years. While we applied standardized case definitions, inconsistencies in medical record-keeping and reporting completeness, particularly in remote areas, could affect the accuracy of incidence and prevalence estimates. Third, the 832 poverty-alleviated counties are not a homogeneous group. Significant internal socioeconomic and ethnic heterogeneity exists within these counties, which our aggregate-level analysis could not dissect. Future studies should incorporate township-level data. Fourth, the comparative analysis between poverty-alleviated and nonpoverty-alleviated regions is constrained by the heterogeneity of data sources. The poverty-alleviated data were extracted from the NHPADMS (a policy-driven active registry), whereas the national comparator data were derived from the GBD 2021 study (a modeling-based estimate). These 2 sources differ substantially in case definitions, case ascertainment completeness, and population coverage. Therefore, absolute prevalence estimates are not directly comparable across the 2 datasets; the present analysis focuses only on comparing the rate of change (APC). No confounding factors (eg, age, sex, comorbidities, socioeconomic status) could be controlled for across the 2 datasets due to their heterogeneous designs. Therefore, readers should interpret any cross-dataset comparison with extreme caution, and only the temporal trend direction (APC) is considered reliable for comparative purposes. Fifth, this study did not differentiate between ischemic and hemorrhagic stroke subtypes. These 2 subtypes have distinct pathophysiological mechanisms, risk factor profiles (eg, hypertension is more strongly associated with hemorrhagic stroke, while atrial fibrillation and dyslipidemia are more linked to ischemic stroke), and treatment strategies. The NHPADMS database does not routinely record stroke subtype information, precluding subtype-specific analysis. Future studies should incorporate stroke subtype data to better tailor prevention and intervention strategies for poverty-alleviated populations.

### Conclusion

This study provides the first systematic evidence that the stroke burden in China’s poverty-alleviated counties is dynamically evolving, characterized by a sharp increase in recorded prevalence, a pronounced shift toward younger adults (aged 20‐39), and distinct geographic clustering in northern regions. These findings indicate a clear disconnect between economic advancement and health gains in the postpoverty transition era. Our high-resolution analysis thus furnishes critical evidence for precision public health, underscoring the imperative to shift interventions toward geographically targeted strategies for identified high-risk clusters and tailored prevention for younger populations to achieve equitable health outcomes.

## Supplementary material

10.2196/91487 Multimedia Appendix 1 Sensitivity analysis of Joinpoint regression trends for stroke incidence and prevalence after excluding 2024 data in poverty-alleviated counties, China.

10.2196/91487 Multimedia Appendix 2 Sensitivity analysis of global spatial autocorrelation (Moran *I*) for stroke incidence across different distance thresholds in poverty-alleviated counties, China (2020-2024).

10.2196/91487 Multimedia Appendix 3 List of counties identified as high-high spatial clusters for stroke incidence, stratified by demographic subgroups, in poverty-alleviated counties of China, 2024.

10.2196/91487 Multimedia Appendix 4 Stroke prevalence in poverty-alleviated regions and China (2019-2021).

## Abbreviations

AAPC: average annual percent change
APC: annual percentage change
ASPR: age-standardized prevalence rate
GBD: Global Burden of Disease
NGCC: National Geomatics Center of China
NHPADMS: National Health Poverty Alleviation Dynamic Management System
WBIC: Weighted Bayesian Information Criterion
