# Supplementary material for: The Evolving Burden of Stroke in China’s 832 Poverty-Alleviated Counties (2019-2024): Nationwide Spatiotemporal Analysis
Source: JMIR Public Health Surveill. 2026 Jun 3;12:e91487. doi: 10.2196/91487 (PMC13232922; doi:10.2196/91487)
Supplement: Multimedia Appendix 1 [file publichealth-v12-e91487-s001.docx]

**Supplementary Table S1.** Sensitivity analysis of Joinpoint regression trends for stroke incidence and prevalence after excluding 2024 data in poverty-alleviated counties, China

| **Year** | **2019** | **2020** | **2021** | **2022** | **2023** | **AAPC ^c^, % (95% CI ^d^)** |
| --- | --- | --- | --- | --- | --- | --- |
| **Incidence** | N/A ^b^ | 107.80 | 99.02 | 102.43 | 104.87 | -0.45 (-5.04 - 4.32) |
| **Incidence by sex** | | | | | | |
| Male | N/A | 113.25 | 105.04 | 109.18 | 112.61 | 0.25 (-4.35 - 5.05) |
| Female | N/A | 101.45 | 92.09 | 99.47 | 96.66 | -0.61 (-4.35 - 3.15) |
| **Incidence by sex** | | | | | | |
| 20-39 | N/A | 2.55 | 1.20 | 2.43 | 2.78 | 10.93 (-21.84 - 56.44) |
| 40-64 | N/A | 79.93 | 70. 91 | 99.75 | 88.99 | 7.02 (-14.46 - 34.08) |
| **≥** 65 | N/A | 384.01 | 367.29 | 380.17 | 426.96 | 3.11 (-7.55 - 13.65) |
| **Prevalence** | 65.18 | 173.11 | 172.79 | 206.63 | 313.74 | 39.34 ^e^ (16.47 - 65.40) |
| **Prevalence by sex** | | | | | | |
| Male | 67.86 | 181.43 | 180.19 | 215.54 | 321.37 | 38.87 ^e^ (16.91 - 63.97) |
| Female | 62.08 | 163.44 | 159.57 | 195.74 | 304.24 | 39.86 ^e^ (14.81 - 68.75) |
| **Prevalence by age** | | | | | | |
| 20-39 | 1.07 | 3.64 | 3.89 | 6.54 | 9.43 | 63.90 ^e^ (34.25 - 98.42) |
| 40-64 | 42.85 | 123.33 | 131.01 | 175.90 | 266.41 | 49.33 ^e^ (22.93 - 80.33) |
| **≥** 65 | 241.48 | 637.86 | 707.68 | 837.58 | 1342.21 | 46.26 ^e^ (16.17 - 77.87) |

Note: This sensitivity analysis was performed to assess whether the sharp decline in stroke incidence in 2024 disproportionately influenced overall trend estimates. Joinpoint regression was repeated using data from 2020 to 2023 (excluding 2024) for incidence and from 2019 to 2023 (excluding 2024) for prevalence. The incidence trend without 2024 remained nonsignificant, confirming that the 2024 decline did not alter the overall conclusion of no sustained increase. The prevalence trend without 2024 remained strongly positive, consistent with the primary analysis.

Abbreviations: ^b^N/A = Not Applicable; ^c^AAPC = Average Annual Percentage Change; ^d^CI = Confidence Interval.

^e^ indicates that the average annual percent change (AAPC) is significantly different from zero at the alpha = 0.05 level. The AAPC in prevalence rates (2019–2023) was statistically significant across all subgroups, although the AAPC for incidence rates (2020–2023) was not.
