# Supplementary material for: The Evolving Burden of Stroke in China’s 832 Poverty-Alleviated Counties (2019-2024): Nationwide Spatiotemporal Analysis
Source: JMIR Public Health Surveill. 2026 Jun 3;12:e91487. doi: 10.2196/91487 (PMC13232922; doi:10.2196/91487)
Supplement: Multimedia Appendix 2 [file publichealth-v12-e91487-s002.docx]

**Supplementary Table S2.** Sensitivity analysis of global spatial autocorrelation (Moran’s I) for stroke incidence across different distance thresholds in poverty-alleviated counties, China (2020–2024)

| **Demographic Subgroup** | **Year** | **Distance Threshold (Meter)** | **Moran’s I** | **Z-score** | ***p*** | **Pattern** |
| --- | --- | --- | --- | --- | --- | --- |
| **Overall** | 2020 | Default (290402) | 0.09 | 11.27 | 0.00 | Clustered |
|  |  | 50% (145201) | 0.13 | 9.35 | 0.00 | Clustered |
|  |  | 200% (580805) | 0.07 | 14.04 | 0.00 | Clustered |
|  | 2021 | Default (290402) | 0.09 | 11.31 | 0.00 | Clustered |
|  |  | 50% (145201) | 0.13 | 9.53 | 0.00 | Clustered |
|  |  | 200% (580805) | 0.06 | 13.43 | 0.00 | Clustered |
|  | 2022 | Default (290402) | 0.19 | 24.31 | 0.00 | Clustered |
|  |  | 50% (145201) | 0.24 | 16.91 | 0.00 | Clustered |
|  |  | 200% (580805) | 0.15 | 30.03 | 0.00 | Clustered |
|  | 2023 | Default (290402) | 0.30 | 35.76 | 0.00 | Clustered |
|  |  | 50% (145201) | 0.42 | 28.44 | 0.00 | Clustered |
|  |  | 200% (580805) | 0.22 | 42.58 | 0.00 | Clustered |
|  | 2024 | Default (290402) | 0.12 | 15.16 | 0.00 | Clustered |
|  |  | 50% (145201) | 0.17 | 11.84 | 0.00 | Clustered |
|  |  | 200% (580805) | 0.09 | 18.11 | 0.00 | Clustered |
| **Ages 20-39** | 2020 | Default (290402) | 0.06 | 7.64 | 0.00 | Clustered |
|  |  | 50% (145201) | 0.07 | 4.86 | 0.00 | Clustered |
|  |  | 200% (580805) | 0.05 | 10.09 | 0.00 | Clustered |
|  | 2021 | Default (290402) | 0.00 | 0.64 | 0.38 | Random |
|  |  | 50% (145201) | 0.01 | 0.74 | 0.46 | Random |
|  |  | 200% (580805) | 0.00 | 1.25 | 0.21 | Random |
|  | 2022 | Default (290402) | 0.03 | 6.65 | 0.00 | Clustered |
|  |  | 50% (145201) | 0.03 | 3.77 | 0.00 | Clustered |
|  |  | 200% (580805) | 0.02 | 8.15 | 0.00 | Clustered |
|  | 2023 | Default (290402) | 0.43 | 53.18 | 0.00 | Clustered |
|  |  | 50% (145201) | 0.68 | 48.18 | 0.00 | Clustered |
|  |  | 200% (580805) | 0.26 | 52.99 | 0.00 | Clustered |
|  | 2024 | Default (290402) | 0.05 | 7.93 | 0.00 | Clustered |
|  |  | 50% (145201) | 0.08 | 7.29 | 0.00 | Clustered |
|  |  | 200% (580805) | 0.03 | 7.50 | 0.00 | Clustered |
| **Ages 40-64** | 2020 | Default (290402) | 0.08 | 10.45 | 0.00 | Clustered |
|  |  | 50% (145201) | 0.12 | 8.11 | 0.00 | Clustered |
|  |  | 200% (580805) | 0.07 | 13.38 | 0.00 | Clustered |
|  | 2021 | Default (290402) | 0.08 | 10.34 | 0.00 | Clustered |
|  |  | 50% (145201) | 0.12 | 8.32 | 0.00 | Clustered |
|  |  | 200% (580805) | 0.06 | 11.74 | 0.00 | Clustered |
|  | 2022 | Default (290402) | 0.29 | 34.88 | 0.00 | Clustered |
|  |  | 50% (145201) | 0.35 | 23.57 | 0.00 | Clustered |
|  |  | 200% (580805) | 0.23 | 43.90 | 0.00 | Clustered |
|  | 2023 | Default (290402) | 0.34 | 40.41 | 0.00 | Clustered |
|  |  | 50% (145201) | 0.48 | 32.51 | 0.00 | Clustered |
|  |  | 200% (580805) | 0.24 | 46.45 | 0.00 | Clustered |
|  | 2024 | Default (290402) | 0.14 | 17.57 | 0.00 | Clustered |
|  |  | 50% (145201) | 0.20 | 14.30 | 0.00 | Clustered |
|  |  | 200% (580805) | 0.09 | 19.05 | 0.00 | Clustered |
| **Ages ≥65** | 2020 | Default (290402) | 0.09 | 11.15 | 0.00 | Clustered |
|  |  | 50% (145201) | 0.10 | 7.49 | 0.00 | Clustered |
|  |  | 200% (580805) | 0.07 | 13.82 | 0.00 | Clustered |
|  | 2021 | Default (290402) | 0.07 | 8.48 | 0.00 | Clustered |
|  |  | 50% (145201) | 0.10 | 7.23 | 0.00 | Clustered |
|  |  | 200% (580805) | 0.05 | 9.54 | 0.00 | Clustered |
|  | 2022 | Default (290402) | 0.37 | 43.64 | 0.00 | Clustered |
|  |  | 50% (145201) | 0.43 | 29.02 | 0.00 | Clustered |
|  |  | 200% (580805) | 0.29 | 56.03 | 0.00 | Clustered |
|  | 2023 | Default (290402) | 0.33 | 39.77 | 0.00 | Clustered |
|  |  | 50% (145201) | 0.47 | 31.85 | 0.00 | Clustered |
|  |  | 200% (580805) | 0.24 | 46.57 | 0.00 | Clustered |
|  | 2024 | Default (290402) | 0.10 | 12.06 | 0.00 | Clustered |
|  |  | 50% (145201) | 0.15 | 10.14 | 0.00 | Clustered |
|  |  | 200% (580805) | 0.07 | 14.16 | 0.00 | Clustered |
| **Male** | 2020 | Default (290402) | 0.09 | 10.98 | 0.00 | Clustered |
|  |  | 50% (145201) | 0.13 | 8.96 | 0.00 | Clustered |
|  |  | 200% (580805) | 0.07 | 13.93 | 0.00 | Clustered |
|  | 2021 | Default (290402) | 0.10 | 11.73 | 0.00 | Clustered |
|  |  | 50% (145201) | 0.15 | 10.11 | 0.00 | Clustered |
|  |  | 200% (580805) | 0.07 | 13.68 | 0.00 | Clustered |
|  | 2022 | Default (290402) | 0.20 | 24.78 | 0.00 | Clustered |
|  |  | 50% (145201) | 0.25 | 17.37 | 0.00 | Clustered |
|  |  | 200% (580805) | 0.15 | 30.65 | 0.00 | Clustered |
|  | 2023 | Default (290402) | 0.32 | 38.19 | 0.00 | Clustered |
|  |  | 50% (145201) | 0.45 | 30.61 | 0.00 | Clustered |
|  |  | 200% (580805) | 0.23 | 45.05 | 0.00 | Clustered |
|  | 2024 | Default (290402) | 0.12 | 14.70 | 0.00 | Clustered |
|  |  | 50% (145201) | 0.16 | 11.58 | 0.00 | Clustered |
|  |  | 200% (580805) | 0.08 | 17.48 | 0.00 | Clustered |
| **Female** | 2020 | Default (290402) | 0.09 | 11.56 | 0.00 | Clustered |
|  |  | 50% (145201) | 0.14 | 9.80 | 0.00 | Clustered |
|  |  | 200% (580805) | 0.07 | 14.03 | 0.00 | Clustered |
|  | 2021 | Default (290402) | 0.08 | 9.99 | 0.00 | Clustered |
|  |  | 50% (145201) | 0.12 | 8.03 | 0.00 | Clustered |
|  |  | 200% (580805) | 0.06 | 12.22 | 0.00 | Clustered |
|  | 2022 | Default (290402) | 0.15 | 19.16 | 0.00 | Clustered |
|  |  | 50% (145201) | 0.19 | 13.20 | 0.00 | Clustered |
|  |  | 200% (580805) | 0.11 | 23.54 | 0.00 | Clustered |
|  | 2023 | Default (290402) | 0.26 | 31.87 | 0.00 | Clustered |
|  |  | 50% (145201) | 0.37 | 25.09 | 0.00 | Clustered |
|  |  | 200% (580805) | 0.20 | 38.48 | 0.00 | Clustered |
|  | 2024 | Default (290402) | 0.12 | 15.46 | 0.00 | Clustered |
|  |  | 50% (145201) | 0.17 | 12.10 | 0.00 | Clustered |
|  |  | 200% (580805) | 0.09 | 18.55 | 0.00 | Clustered |

Notes: Spatial weights were constructed using the INVERSE DISTANCE conceptualization in ArcGIS 10.8. The default distance threshold was automatically determined by ArcGIS as 290,402 meters. Sensitivity thresholds were set to 50% (145,201 m) and 200% (580,804 m) of the default value. For each threshold and each year, the Global Moran’s I index, z-score, and associated p-value are reported. A statistically significant positive Moran’s I (p < 0.05) indicates spatial clustering of similar incidence values. The results demonstrate that the magnitude, direction, and significance of spatial autocorrelation remain consistent across all three thresholds, confirming the robustness of the spatial clustering findings.
