# Supplementary material for: The Evolving Burden of Stroke in China’s 832 Poverty-Alleviated Counties (2019-2024): Nationwide Spatiotemporal Analysis
Source: JMIR Public Health Surveill. 2026 Jun 3;12:e91487. doi: 10.2196/91487 (PMC13232922; doi:10.2196/91487)
Supplement: Multimedia Appendix 3 [file publichealth-v12-e91487-s003.docx]

**Supplementary Table S3.** List of counties identified as high-high (HH) spatial clusters for stroke incidence, stratified by demographic subgroups, in poverty-alleviated counties of China, 2024.

| **Demographic Subgroup** | **HH Clusters** | |
| --- | --- | --- |
|  | **Province** | **County** |
| **Overall** | | |
|  | Hebei | Wei County, Daming County, Lincheng County, Xinhe County, Julu County, Pingxiang County, Wei County, Guangzong County, Zanhuang County, Raoyang County, Wuyi County, Fucheng County, Nanpi County, Yanshan County, Haixing County, Pingshan County, Lingshou County, Xingtang County, Fuping County, Quyang County, Tang County, Wangdu County, Laiyuan County, Yi County, Laishui County, Yu County, Yangyuan County, Huaian County, Shangyi County, Wanquan District, Zhangbei County, Chongli District, Kangbao County, Chicheng County, Guyuan County, Fengning Manchu Autonomous County, Weichang Manchu and Mongolian Autonomous County, Longhua County, Luanping County, Chengde County |
|  | Heilongjiang | Fuyuan City, Tangyuan County, Fuyu County, Longjiang County, Tailai County |
|  | Inner Mongolia | Jalaid Banner, Taibus Banner, Zhengxiangbai Banner |
|  | Anhui | Si County, Huoqiu County, Yu'an District, Yingshang County |
|  | Yunnan | Linxiang District |
| **Ages 20–39** | | |
|  | Hebei | Wei County, Lincheng County, Xinhe County, Julu County, Pingxiang County, Wei County, Zanhuang County, Wuqiang County, Pingshan County, Lingshou County, Fuping County, Quyang County, Laiyuan County, Yi County, Laishui County, Yu County, Shunping County |
|  | Yunnan | Linxiang District, Menghai County, Gengma Dai and Va Autonomous County, Yongde County, Yun County, Menglian Dai, Lahu and Va Autonomous County |
|  | Shanxi | Wutai County |
| **Ages 40-64** | | |
|  | Hebei | Wei County, Daming County, Xinhe County, Julu County, Pingxiang County, Wei County, Guangzong County, Raoyang County, Wuyi County, Fucheng County, Nanpi County, Yanshan County, Haixing County, Pingshan County, Lingshou County, Xingtang County, Fuping County, Quyang County, Tang County, Wangdu County, Laiyuan County, Yi County, Laishui County, Yu County, Yangyuan County, Huaian County, Shangyi County, Wanquan District, Zhangbei County, Chongli District, Kangbao County, Guyuan County, Fengning Manchu Autonomous County, Weichang Manchu and Mongolian Autonomous County, Longhua County, Shunping County |
|  | Heilongjiang | Fuyuan City, Tangyuan County |
|  | Inner Mongolia | Taibus Banner, Zhengxiangbai Banner |
|  | Anhui | Si County, Huoqiu County, Shou County |
|  | Yunnan | Linxiang District, Gengma Dai and Va Autonomous County, Yongde County, Yun County, Menglian Dai, Lahu and Va Autonomous County |
| **Ages ≥65** | | |
|  | Hebei | Wei County, Daming County, Lincheng County, Xinhe County, Julu County, Pingxiang County, Wei County, Guangzong County, Zanhuang County, Raoyang County, Wuyi County, Nanpi County, Haixing County, Pingshan County, Lingshou County, Xingtang County, Fuping County, Quyang County, Tang County, Wangdu County, Laiyuan County, Yi County, Laishui County, Yu County, Yangyuan County, Huaian County, Shangyi County, Wanquan District, Zhangbei County, Chongli District, Kangbao County, Chicheng County, Guyuan County, Fengning Manchu Autonomous County, Weichang Manchu and Mongolian Autonomous County, Longhua County, Luanping County, Chengde County |
|  | Heilongjiang | Raohe County, Fuyu County, Longjiang County, Tailai County |
|  | Inner Mongolia | Jalaid Banner, Taibus Banner, Zhengxiangbai Banner |
|  | Anhui | Si County, Huoqiu County, Yu'an District |
|  | Yunnan | Linxiang District, Gengma Dai and Va Autonomous County, Yongde County, Menghai County, Menglian Dai, Lahu and Va Autonomous County |
|  | Hubei | Yunyang District |
| **Male** | | |
|  | Hebei | Wei County, Daming County, Lincheng County, Xinhe County, Julu County, Pingxiang County, Wei County, Guangzong County, Raoyang County, Nanpi County, Yanshan County, Haixing County, Pingshan County, Lingshou County, Xingtang County, Fuping County, Quyang County, Tang County, Wangdu County, Laiyuan County, Yi County, Laishui County, Yu County, Yangyuan County, Huaian County, Shangyi County, Wanquan District, Zhangbei County, Chongli District, Kangbao County, Chicheng County, Guyuan County, Fengning Manchu Autonomous County, Weichang Manchu and Mongolian Autonomous County, Longhua County, Luanping County, Chengde County |
|  | Heilongjiang | Fuyuan City, Tangyuan County, Longjiang County, Tailai County |
|  | Inner Mongolia | Jalaid Banner, Taibus Banner, Zhengxiangbai Banner |
|  | Anhui | Si County, Huoqiu County, Yu'an District, Yingshang County, Shou County |
| **Female** | | |
|  | Hebei | Wei County, Daming County, Lincheng County, Xinhe County, Julu County, Pingxiang County, Wei County, Guangzong County, Zanhuang County, Raoyang County, Wuyi County, Fucheng County, Nanpi County, Yanshan County, Haixing County, Pingshan County, Lingshou County, Xingtang County, Fuping County, Quyang County, Tang County, Wangdu County, Laiyuan County, Yi County, Laishui County, Yu County, Yangyuan County, Huaian County, Shangyi County, Wanquan District, Zhangbei County, Chongli District, Kangbao County, Chicheng County, Guyuan County, Fengning Manchu Autonomous County, Weichang Manchu and Mongolian Autonomous County, Longhua County, Luanping County, Chengde County, Qinglong Manchu Autonomous County |
|  | Heilongjiang | Raohe County, Longjiang County, Tailai County |
|  | Inner Mongolia | Jalaid Banner, Taibus Banner, Zhengxiangbai Banner |
|  | Anhui | Si County, Huoqiu County, Lixin County |
|  | Yunnan | Linxiang District, Gengma Dai and Va Autonomous County |

Note: This table lists county-level administrative units identified as statistically significant High-High (HH) clusters for stroke incidence within each demographic subgroup. An HH cluster indicates a county with a high incidence rate that is surrounded by other counties with similarly high rates (i.e., a hotspot), as categorized by the Local Indicators of Spatial Association (LISA) analysis using Anselin Local Moran’s I.

Spatial weights: A first-order Queen contiguity matrix (neighbors sharing a boundary or vertex) was used to model spatial relationships.

Statistical significance: The significance of clusters was determined using a permutation approach with 999 repetitions at a significance level of p < 0.05.

Analysis software: All spatial autocorrelation analyses were performed at the county level using ArcGIS 10.8.

Data source: China's National Health Poverty Alleviation Dynamic Management System, 2024.
