# Supplementary material for: The Evolving Burden of Stroke in China’s 832 Poverty-Alleviated Counties (2019-2024): Nationwide Spatiotemporal Analysis
Source: JMIR Public Health Surveill. 2026 Jun 3;12:e91487. doi: 10.2196/91487 (PMC13232922; doi:10.2196/91487)
Supplement: Multimedia Appendix 4 [file publichealth-v12-e91487-s004.docx]

**Supplementary Table S4.** Stroke Prevalence in Poverty-Alleviated Regions and China (2019-2021)

| **Region** | **Overall** | | | **Ages 20–39** | | **Ages 40–64** | | **Ages ≥65** | |
| --- | --- | --- | --- | --- | --- | --- | --- | --- | --- |
|  | **2021 PR ^a^** | **2021 ASPR ^b^**  **(95% CI)** | **2019–2021 APC^c^**  **(95% CI ^d^)** | **2021 PR** | **2019–2021 APC**  **(95% CI)** | **2021 PR** | **2019–2021 APC**  **(95% CI)** | **2021 PR** | **2019–2021 APC**  **(95% CI)** |
| China (overall) | 1851.03 | 1298.30  (1298.29-1298.32) | 1.64^e^  (0.32-3.03) | 422.68 | 1.78 ^e^  (1.09-2.47) | 1886.92 | 1.57 ^e^  (0.67-2.50) | 7555.38 | 1.73 ^e^  (0.04-3.62) |
| Poverty- alleviated regions | 172.79 | 96.42  (96.40-96.44) | 61.85 ^e^  (14.99-129.60) | 3.89 | 88.96 ^e^  (26.77-184.81) | 131.01 | 73.62 ^e^  (22.90-147.48) | 707.68 | 74.10 ^e^  (26.97-136.55) |
| Non-poverty-alleviated regions | 1919.92 | 1317.11  (1316.46-1317.32) | 3.82 ^e^  (2.31-5.40) | 441.91 | 1.94 ^e^  (1.11-2.77) | 1953.64 | 1.57 ^e^  (0.52-2.65) | 7930.07 | 0.96  (-0.72-2.83) |
| *χ^2^* | 905,600^f^ | / | / | 292,600^f^ | / | 319,400^f^ | / | 720,500^f^ | / |

Note: The group labelled “Non-poverty-alleviated regions” refers to data for China excluding 832 designated poverty-alleviated regions. Prevalence rates were age-standardized using the GBD 2021 reference population structure (Institute for Health Metrics and Evaluation) to ensure comparability.

Abbreviations: ^a^PR = Prevalence Rate (/100,000); ^b^ASPR = Age-Standardized Prevalence Rate (/100,000); ^c^APC = Annual Percentage Change; ^d^CI = Confidence Interval.

^e^ denotes a statistically significant annual trend, with a p-value < 0.01 for the Annual Percentage Change (APC). The APC and its 95% confidence interval were calculated using Joinpoint Regression (National Cancer Institute, Version 5.2.0). P < 0.01 indicates a statistically significant annual trend in prevalence. As the model failed to detect the inflection point, the Average Annual Percentage Change (AAPC) and APC values are identical.

^f^ indicates that the result of the chi-square test is statistically significant at *p* < 0.001. This test was used to compare prevalence rates in 2021 between poverty-alleviated regions and non-poverty-alleviated regions, with the between-group difference being statistically significant.
